# Does Adipose Tissue Thermogenesis Play a Role in Metabolic Health?

**DOI:** 10.1155/2013/204094

**Published:** 2013-04-17

**Authors:** Craig Porter, Elisabet Børsheim, Labros S. Sidossis

**Affiliations:** ^1^Metabolism Unit, Shriners Hospitals for Children, Department of Surgery, University of Texas Medical Branch, Galveston, TX 77550, USA; ^2^Department of Surgery, University of Texas Medical Branch, Galveston, TX 77550, USA; ^3^Department of Internal Medicine, Sealy Center on Aging, Institute for Translational Sciences, University of Texas Medical Branch, Galveston, TX 77550, USA; ^4^Department of Nutrition and Dietetics, Harokopio University, Athens, Greece

## Abstract

The function ascribed to brown adipose tissue in humans has long been confined to thermoregulation in neonates, where this thermogenic capacity was thought lost with maturation. Recently, brown adipose tissue depots have been identified in adult humans. The significant oxidative capacity of brown adipocytes and the ability of their mitochondria to respire independently of ATP production, has led to renewed interest in the role that these adipocytes play in human energy metabolism. In our view, there is a need for robust physiological studies determining the relationship between molecular signatures of brown adipose tissue, adipose tissue mitochondrial function, and whole body energy metabolism, in order to elucidate the significance of thermogenic adipose tissue in humans. Until such information is available, the role of thermogenic adipose tissue in human metabolism and the potential that these adipocytes may prevent or treat obesity and metabolic diseases in humans will remain unknown. In this article, we summarize the recent literature pertaining to brown adipose tissue function with the aims of drawing the readers' attention to the lack of data concerning the role of brown adipocytes in human physiology, and to the potential limitations of current research strategies.

## 1. Introduction

As the combustion engine of respiring cells, the mitochondrion is an essential component of life. Furthermore, as these organelles are principally responsible for the oxidative disposal of glucose and fatty acids, the role of mitochondrial function in the etiology of diseases where substrate metabolism is perturbed has been the focus of a considerable research effort for a number of decades. A notable example of this is the role of mitochondrial capacity in the pathogenesis of skeletal muscle insulin resistance. The reason for such interest in the role of altered skeletal muscle bioenergetics in the development of insulin resistance are several fold; skeletal muscles significant contribution to body mass, the relative abundance of mitochondria within skeletal muscle and their plasticity (particularly in response to muscular contraction), skeletal muscles central role in whole body glucose and fatty acid disposal, and the fact that skeletal muscle can be sampled from humans relatively easily are all contributing factors. With regard to skeletal muscle, whether insulin resistance is indeed the chicken, with mitochondrial dysfunction being the preceding egg, or whether the opposite is the case remains unclear. However, what does seem clear is that measures of skeletal muscle oxidative capacity and mitochondrial content correlate well with skeletal muscle insulin sensitivity [1, 2], making them attractive outcome measures for researchers interested in insulin sensitivity.

In contrast to skeletal muscle, adipose tissue has long been considered a relatively quiescent tissue in humans, particularly with regards to its oxidative metabolism, where it was generally thought to function as a storage depot for excess calories. While several depots of adipose tissue are present in humans (including but not limited to subcutaneous, visceral, epicardial, and brown), the two major stores, subcutaneous and visceral, both have the capacity to significantly expand in obese subjects, where it has been proposed that these two adipose tissue depots contribute differently to metabolic risk factors in humans [3]. However, as adipose tissue has far fewer mitochondria than other tissues such as liver and muscle, the role of adipose tissue mitochondrial function (if any) in human physiology has been paid very little attention to date. The principal reason for this is that, until recently, the prevailing dogma was that brown adipose tissue was only found in small animals and in human neonates, where it played a role in nonshivering thermogenesis. However, as brown adipose tissue has now been identified in adult humans [4–7], and perhaps just as intriguingly, the identification of UCP1 positive adipocytes within white adipose tissue [8], has led to renewed interest in the scientific community as to whether metabolically active (thermogenic) adipose tissue depots play a significant role in human physiology.

## 2. Adipose Tissue Mitochondrial Function

Interest in adipose tissue mitochondria pertains not to their role in ATP production, but rather their ability to respire independently of ATP production, that is, thermogenesis. While this process may be considered detrimental, particularly in organs with high ATP turnover rates like the heart, it allows mitochondria to produce heat as opposed to chemical energy, a significant process in mammalian thermoregulation. Unlike other tissues, brown adipocytes contain uncoupling protein 1 (UCP1), which, despite having homologues (UCP2–5) in other tissues, is the only UCP thought to have the ability to significantly uncouple the respiratory chain when activated *in vivo*. In this instance, UCP1 essentially short-circuits the electron transport chain, meaning that the intramembrane proton gradient of the mitochondrion is dissipated independently of ATP synthase. Indeed, the chemiosmotic theory of oxidative phosphorylation put forward by Mitchell [9] described how the excursion of protons from the matrix of the mitochondrion to its intramembrane space results in the formation of a proton gradient, which dissipates when protons re-enter the mitochondrial matrix via complex V of the respiratory chain, ATP synthase. ATP synthase harnesses this potential energy and uses it to phosphorylate ADP, thus producing ATP. Interestingly though, as per the laws of thermodynamics, when the electron transport chain is uncoupled, this chemical energy is not lost, rather it is dissipated as heat. As such, UCP1 was classically described as thermogenin, due to its ability to generate heat from mitochondrial respiration [10]. While the physiological role ascribed to the uncoupling of mitochondrial respiration from ATP synthesis is thermoregulation, renewed interest in the ability of UCP1 positive adipocytes to produce heat and thus increase energy expenditure relates to the potential that activation of this tissue may alter energy expenditure and body mass in humans.

## 3. Can UCP1 Positive Adipocytes Alter Metabolism in Humans?

The relatively recent identification of brown adipose tissue depots in humans has led to renewed interest in the scientific community as to whether metabolically active (thermogenic) adipose tissue plays a role in human body weight regulation and metabolism [11]. In our view, the two most apparent questions that remain unanswered are as follows. (1) What are the physiological roles of brown and brown-like adipocytes and (2) does the activation of brown fat *in vivo* in humans alter metabolism (basal metabolic rate, glucose disposal, and lipid profile)?

Experimentally, an abundance of data derived from murine models has evidenced that brown adipose tissue activation can alter energy metabolism *in vivo* [12, 13]. However, a broader caveat of the use of rodents, and particularly mice in metabolic research, relates to mammalian thermoregulation. In general, animal rooms in research facilities are kept at temperatures that are ambient to human beings (close to ~20°C). However, as discussed in detail by Cannon and Nedergaard [14], a thermoneutral temperature for a rodent (particularly young mice) is around 30°C. This poses a problem as it suggests that the majority of rodents used in research may be cold and thus have chronically activated brown fat. As such, this consideration makes the translation of these animal studies to human metabolism questionable. 

Brown adipose tissue, with its high mitochondrial density and oxidative capacity, is more akin to skeletal muscle than white adipose tissue [15]. While skeletal muscles oxidative capacity, that is, its ability to respire and produce ATP, is considerable, this potential is only realized during near maximal muscular contraction, when ATP turnover rates increase considerably. This begs the question of whether a tissue with a similar metabolic potential, such as brown adipose tissue, can be periodically or even chronically activated in humans to an extent that significantly alters energy expenditure. Given brown adipose tissues role in thermogenesis, investigators typically expose rodents to prolonged cold exposure to demonstrate activation of brown adipose tissue [10], an approach which also works in humans [7]. However, in removing the physiological stimulus (cold exposure), one would assume then that brown adipose tissue would return to a more quiescent state. Indeed, returning cold exposed rodents to thermoneutral conditions attenuates brown adipose tissue activation, suggesting that a chronic physiological stimulus is obligatory for UCP1 activation. Moreover, it appears that UCP1 is not spontaneously leaky, being inhibited *in vivo* by purine nucleotides [16], suggesting that having brown adipose tissue alone may not alter energy expenditure. 

While recent research has indeed confirmed the presence of brown adipocytes in humans, the relationship between these UCP1 positive adipocytes and energy expenditure, particularly in relation to clinical outcomes such as obesity and insulin sensitivity, remains to be seen. A limitation of the current literature is the reliance on a UCP1 signal to infer thermogenesis *in vivo *[17]. While the presence of UCP1 mRNA in adipocytes does indeed suggest a greater oxidative capacity and, importantly, a greater capacity for uncoupled respiration, measurement of UCP1 mRNA expression alone says little about the activation of the mitochondrion or UCP1 *in vivo*. Subsequently, concurrent measures of energy metabolism and adipose tissue bioenergetics will likely play an important role in elucidating the significance of UCP1 positive fat in human physiology. With that said, careful consideration into the determination of mitochondrial function in adipocytes is needed in order to ensure that the best representation of function *in vivo* is maintained in *ex vivo/in vitro* analyses. For example, with regards to skeletal muscle mitochondrial function in insulin resistance, varied outcome measures used as markers of mitochondrial function and content employed by different laboratories have muddied the waters with regards to forming a scientific consensus [18]. Moreover, it would seem that determining mitochondrial function *in vitro* in isolated organelles versus organelles which remain *in situ* also may influence experimental results [19–21]. This may be of particular relevance to UCP1 positive adipocytes given that the UCP1 of isolated mitochondria may not be under the same level of inhibition *in vitro* as would be the case *in vivo *[22].

While data concerning bioenergetics in adipose tissue are scarce, subcutaneous adipose tissue mitochondrial respiration was first determined by Hallgren and colleagues [23]. These investigators suggested that mitochondrial respiration in adipose tissue contributed to ~4% of calculated 24 hr resting energy expenditure. In addition, they also reported that obesity and advancing age were associated with a decline in adipose respiration per gram of tissue [23]. More recently, Kraunsøe and co-workers [24] demonstrated that visceral adipose tissue contained significantly more mitochondria than subcutaneous adipose tissue from obese humans. Moreover, visceral adipose tissue mitochondria were more sensitive to substrates than their counterparts in subcutaneous adipose tissue [24]. Interestingly, the above findings suggest that adipose tissue bioenergetics are influenced by age and obesity, and that mitochondrial density and intrinsic function differs in different adipose tissue depots. While the clinical significance of these findings remain unresolved, evidence from severely burned patients suggests that brown adipose tissue may play a role in the hypermetabolic response associated with this type of trauma. For example, the marked increase in energy expenditure following thermal injury can only partly be accounted for by ATP consuming reactions, where uncoupled mitochondrial respiration has been suggested to contribute to hypermetabolism in severely burned individuals [25]. Indeed, in rodents at least, UCP1 mRNA is induced in both brown and white adipocytes following burn injury [26, 27]. 

## 4. Is the Future “Brite” for Brown Adipose Tissue?

The recent finding that humans contain brown adipose tissue depots and that thermogenic adipocytes may also reside in white adipose tissue depots has led to renewed interest in the role that UCP1 positive adipocytes play in human metabolism [28]. The supposition that these adipocytes, which are more comparable to skeletal muscle with regards to oxidative capacity, may significantly alter energy expenditure in humans via uncoupled mitochondrial respiration is a reasonable one. For example, assuming that respiration in adipose tissue accounts for ~4% of resting metabolic rate [23], or approximately 100 kcal per day in an adult requiring 2500 kcal a day to maintain body mass, then a significant increase in the adipose tissue respiration (in the form of uncoupled respiration) over several months or even years could theoretically have a meaningful impact on body mass, adiposity, and thus metabolic health. Given the current proportion of children and adults affected by the metabolic complications associated with obesity, particularly in the developed world, the therapeutic potential of harnessing the thermogenic properties of UCP1 positive adipocytes is of great interest to both biomedical researchers and the pharmaceutical industry. However, for this potential to be realized, immediate questions as to how UCP1 positive adipocytes can be induced and/or activated* in vivo *in free living humans needs to be addressed. Detailed studies of both whole body metabolism and adipose tissue mitochondrial function in animals maintained in thermoneutral conditions, as well as in healthy humans and patient cohorts, will be central to this process.
